# Case Report: Challenging Perioperative Decision-Making in a Neonate With Transposition of Great Arteries and Novel Coronary Anatomy

**DOI:** 10.3389/fped.2022.900142

**Published:** 2022-07-06

**Authors:** Madhuradhar Chegondi, Marco Ricci, Ravi C. Ashwath

**Affiliations:** ^1^Division of Critical Care Medicine, Stead Family Children's Hospital & Department of Pediatrics, Carver College of Medicine, University of Iowa, Iowa City, IA, United States; ^2^Division of Pediatric Cardiac Surgery, Stead Family Children's Hospital, Iowa City, IA, United States; ^3^Division of Pediatric Cardiology, Stead Family Children's Hospital, Iowa City, IA, United States

**Keywords:** novel, coronary artery pattern, intramural course, commissural malalignment, transposition of great arteries (TGA), neonate, perioperitave decision

## Abstract

Transposition of great arteries (d-TGA) is often associated with various coronary artery (CA) patterns. These anomalous patterns can cause variable clinical symptoms of coronary ischemia including sudden death. CA pattern is one of the major determinants of outcome in TGA postoperatively. An advanced cardiac imaging and a multidisciplinary care approach are essential for a favorable outcome. Here, we describe a novel CA origin pattern in a neonate with d-TGA, who developed myocardial ischemia and required a coronary unroofing procedure for a full recovery.

## Introduction

Various coronary artery (CA) origin patterns are known to occur with transposition of great arteries (d-TGA) (1). In addition, CA anomalies such as intramural course and commissural malalignment (CM) are important factors to be determined preoperatively for the optimal surgical outcome (2). We report a neonate with d-TGA with a novel CA pattern, whose post-operative course was complicated due to a single aortic sinus origin of CAs with an intramural course and CM requiring an unroofing procedure.

## Case Presentation

**Figure 1:**
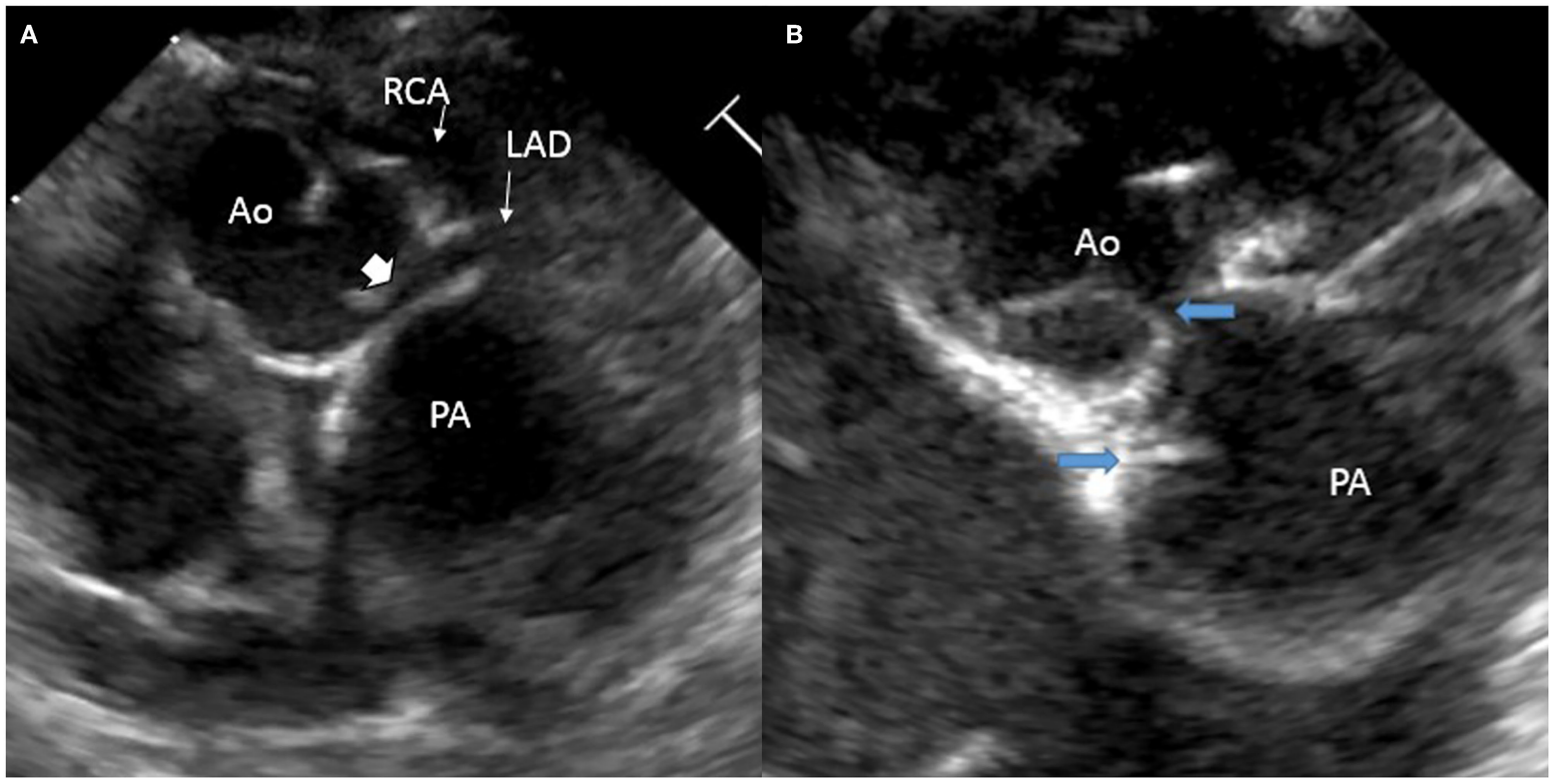
Echocardiogram images: **(A)** intramural course (solid arrowhead) of the main segment of the coronary artery that divides into the right coronary artery (RCA) and the left anterior descending (LAD) from the posterior sinus (Sinus 1); **(B)** commissural malalignment as indicated by the blue arrows.

A full-term baby boy with a prenatal diagnosis of d-TGA was born *via* C-section due to a non-reassuring fetal heart rate. The pregnancy was complicated by maternal hypothyroidism and anemia. His birth weight was 2.9 kg and the Apgar scores were 8 at 1 min and 9 at 5 min. On ambient air, his oxygen saturation (SPO2) was 80%. He had a grade II/VI systolic murmur over the precordium. Intravenous access was established and started on low-dose prostaglandin infusion to maintain ductal patency while obtaining a transthoracic echocardiogram (TTE). The TTE confirmed d-TGA with an intact ventricular septum, a large patent ductus arteriosus (PDA) with a bidirectional shunt, and an unrestrictive atrial level communication. Additional findings on TTE included an intramural course of the main segment of the CA that divided into the right CA (RCA) and the left anterior descending (LAD) from the posterior sinus (Sinus 1) with CM (Figure 1). There was also a suspicion of retro-pulmonary left circumflex CA with its origin not well defined.

**Figure 2:**
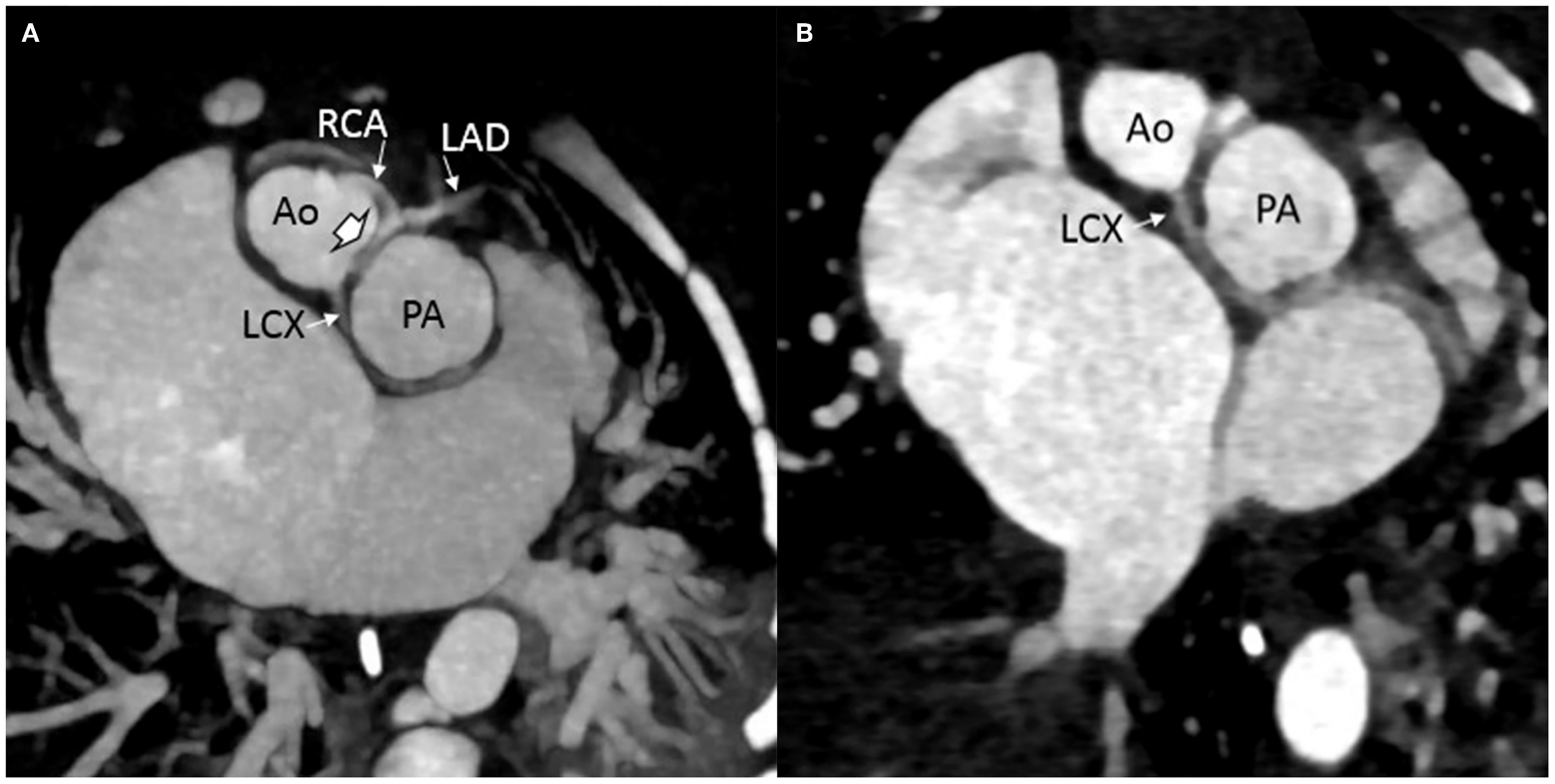
Computed tomography images showing preoperative arrangement of the coronary artery pattern: **(A)** intramural course (solid arrowhead) of the main segment of the coronary artery that divides into the right coronary artery (RCA) and the left anterior descending (LAD) from the posterior sinus (Sinus 1); left circumflex coronary artery (LCX) seen arising from the same sinus. **(B)** LCX seen arising from the Sinus 1 with a retro-pulmonary course.

Once the unrestrictive nature of the atrial septum was confirmed, prostaglandin was discontinued. A cardiac CT angiogram (CTA) was obtained to delineate the coronary anatomy further. The CTA showed an unusual novel CA pattern with two separate ostia, one of which was a main CA segment which is divided into RCA and LAD, and the other was left circumflex with a retro-pulmonary course, both of which arose from the right posterior aortic sinus (Sinus 1). The main segment had an inter arterial course and there was a suggestion of a possible intramural course (Figure 2). The baby was felt to be a good candidate for an arterial switch operation (ASO).

An intraoperative inspection of the coronary arteries confirmed the CT findings. The first coronary ostium originating from the aorta (carrying the LAD and RCA) was located high in the root at the level of the sino-tubular junction and was juxta-commissural. After careful inspection, the ostium did not appear to have an intramural portion and did not appear to be obstructed nor did it have the “slit” appearance as an intramural CA would have. Therefore, the decision was made not to proceed with unroofing, as unroofing in the absence of intramurality would have resulted in injury to the CA and possibly going outside the aorta. Also, the CA transfer with two separate buttons was not feasible, since both ostia were arising from the same sinus and were in close proximity to each other (approximately 2 mm), thereby preventing separation of the ostia into two different CA buttons. The presence of CM between the facing commissures of the aorta and the pulmonary valves further complicates the anatomical findings. Hence, the coronary arteries were transferred using a Yacoub aortocoronary flap-over technique, where the coronary arteries are transferred as a single button, minimizing torsion and rotation, thereby minimizing the risk of coronary obstruction, as previously described by others (3). This technique also allowed the transfer of the single button into the anterior facing sinus of the aorta, away from the commissural posts of the neo-aortic valve, due to the presence of the previously described CM which in a way facilitated the transfer as a single button.

**Figure 3:**
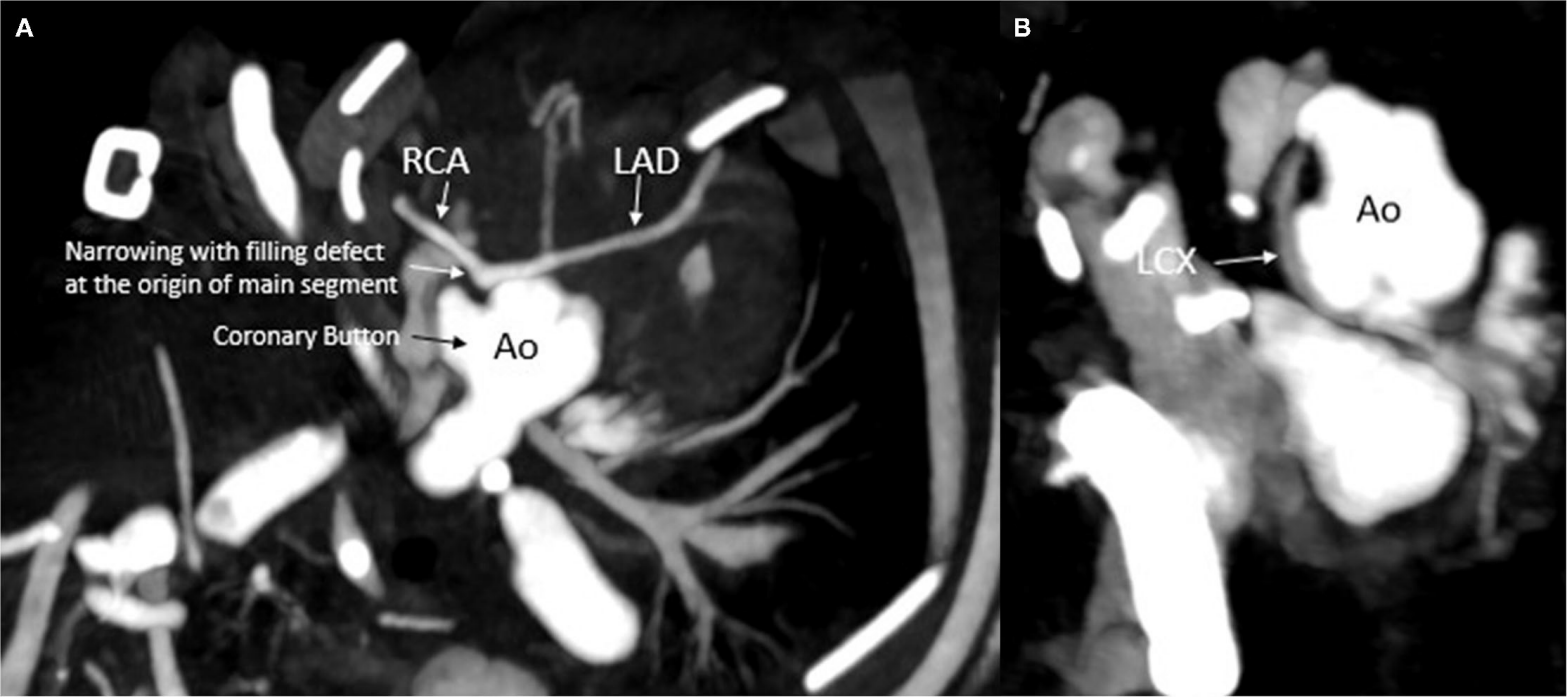
Computed tomography depicting the coronary arteries after initial surgery: **(A)** narrowing of the main segment. **(B)** Normal left circumflex filling with no obvious narrowing. Ao, aorta; RCA, right coronary artery; LAD, left anterior descending coronary artery; LCX, left circumflex coronary artery.

**Figure 4:**
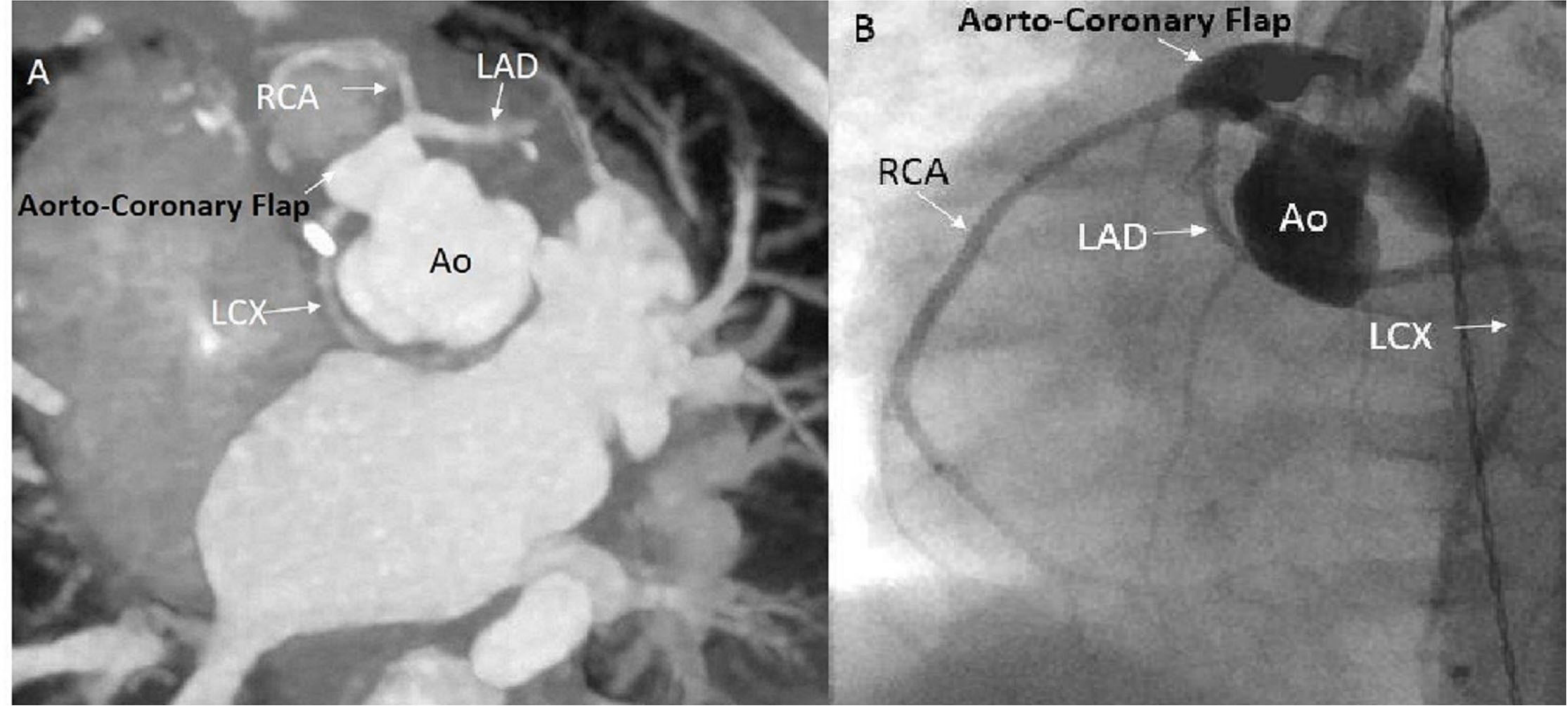
Computed tomography angiogram (CTA) and routine angiogram images obtained after the un-roofing procedure. **(A)** CTA showing the aorto-coronary flap with widely patent coronary arteries. **(B)** Angiogram showing the aorto-coronary flap with widely patent coronary arteries. Ao, Aorta; RCA, right coronary artery; LAD, left anterior descending coronary artery; LCX, left circumflex coronary artery.

During the rewarming, biventricular dysfunction was noted, likely due to intermittent coronary insufficiency. Because the RCA looped around the neo-pulmonary artery anteriorly, the main pulmonary artery was relocated not connecting it to the bifurcation but more toward the right pulmonary artery to avoid undue tension on the right coronary. This, however, did not seem to improve the function significantly. The patient was placed on venous arterial ECMO (VA ECMO) with suspicion of continued coronary insufficiency. The patient was taken to the cardiac catheterization laboratory for coronary angiography with root injection and not selective angiography, which demonstrated good flow to all the coronary arteries. The baby was transferred back to the pediatric cardiac intensive care unit (PCICU) on VA ECMO in stable condition. The next day, follow-up Trans-esophageal echocardiogram (TEE) at the bedside showed continued poor left ventricular function while on VA ECMO and a dyskinetic ventricular septum. There was a strong suspicion of ongoing coronary ischemia pertaining to the LAD and RCA territory. Even though the coronary angiography excluded the presence of the major coronary obstruction and demonstrated good flow in the three coronary arteries, we decided to obtain a repeat cardiac CTA with contrast to further delineate the coronary anatomy. The CTA revealed evidence of obstructed flow in the proximal portion of the main segment of the coronary carrying the LAD and RCA (Figure 3). The patient was taken back to the operating room for CA revision. Upon surgical exploration, we proceeded by unroofing the ostium carrying the RCA and LAD and extended the coronary arterioplasty down to the bifurcation between RCA and LAD as the entire coronary trunk appeared stenotic in its entire course. The entire coronary trunk was then patched with a native pericardial patch, obtaining a complete arterioplasty of the coronary trunk. Post-procedure TEE demonstrated globally reduced but improved left ventricular function as compared to pre-operative echocardiogram; also, of importance, the ventricular septum was no longer dyskinetic and appeared to have regained contractility. A direct visualization of the epicardial coronary arteries demonstrated that the coronary arteries appeared full on the surface of the heart. The patient was taken back to the PCICU on full VA ECMO support. The following day, a TTE showed significant improvement of the biventricular systolic function with satisfactory septal motion. The child was weaned successfully from ECMO support and had an uneventful post-operative recovery. He was discharged home with normal ventricular function and unobstructed flow into the coronary arteries. The patient is currently 6 months old and thriving well with good flow seen in the coronary arteries by TTE. A follow-up CTA and coronary angiogram demonstrated adequate filling of the coronary arteries with an unobstructed flow (Figure 4).

## Discussion

Transposition of great arteries can be associated with various CA patterns (2). These major CA courses could be normal, looping, or intramural (4). In contrast to a normal heart, in TGA, the coronary sinus is more posterior, and the non-coronary sinus is anterior (1). Similarly, the side-to-side relation of great arteries is associated with an increased prevalence of various CA patterns (5). These CA patterns are classified according to their epicardial configuration at the base of the heart (5). The CA pattern is one of the major determinants of outcome in TGA ever since ASO became the surgical procedure of choice (2, 6). In TGA, the most common CA pattern is left main CA originating from the left anterior sinus then dividing into LAD and LCx and RCA arising from the right posterior sinus and the least common being LCA, RCA arising from the two separate ostia of the left anterior sinus with the intramural course of RCA (1).

What made our patient and our operative experience worth reporting, in our opinion, is based on several factors. These included the following. (1) The novel CA arrangement; (2) the difficulty in being very certain about the intramural course with echocardiography alone; (3) the intra-operative decision-making process; (4) the pitfalls of post-operative coronary angiography in detecting coronary obstruction and the usefulness of CTA in defining CA anatomy post-arterial switch; (5) the utility of coronary arterioplasty with native pericardium in the management of obstructed coronary arteries in the setting of ASOs.

Our patient presented with a novel CA pattern in terms of origin and course, with the main CA (MCA) carrying the RCA and LAD and LCx originating from the right posterior sinus with two separate ostia with an intramural course of the MCA segment. The LCx took a retro-pulmonary course. This combination of the CA pattern, to our knowledge, is novel and has not been described previously as per our extensive literature search.

Similar to our case, CM between the semilunar valves is associated with various CA patterns and is a poor prognostic factor in ASO outcomes (7, 8). Depending on the degree of CM, CAs may be subjected to torsion and can get stretched during ASO (7). To avoid the resulting myocardial ischemia, the patient may need an extensive dissection of CAs, trap door incision, and supra or juxta commissural CA transfer (7, 8).

Previously, CA anomalies were diagnosed only on autopsy; however, the advancement of imaging modalities such as CTA and cardiac MRI has enabled diagnosis of various CA patterns, their complete assessment, and reduced mortality with pre-planning (2, 9, 10). The TTE is the imaging modality used most frequently to assess the CA anatomy in neonates with TGA. However, the presence of an intramural coronary course can be missed by TTE, resulting in increased perioperative mortality (10, 11).

The presence of an intramural course of CA has been shown to be a predictor of increased post-ASO morbidity and mortality (2, 3, 12, 13). Moll et al. in their retrospective study, reported significantly higher mortality with intramural CA compared to other coronary variants (27.3 vs. 8.7%; *p* = 0.02) (12). However, a long-term follow-up among the survivors with intramural CA did not develop any complications or require reintervention (3, 14). In a meta-analysis, Pasquali et al. (2) reported a more than 6-fold increase in mortality in patients with an intramural course of CA undergoing ASO. The presence of intramural CA poses a significant technical challenge during the CA transfer, as it may cause injury to the intramural CA while dividing it from the aortic wall. In addition, intramural CA often originates from the wrong aortic sinus and is located very close to the facing commissure, and this requires part of facing commissural removal during the CA separation from the aortic wall (14). Similarly, since our patient's CA ostia were very close, he needed a single-button technique to preserve the proximal CA portion in the normal anatomical position and minimize CA torsion. Sun et al. reported favorable postoperative results using the double coronary button technique for coronary transfer with unroofing of the intramural course (13). Intraoperatively, the insertion of a coronary probe into the intramural portion of CA is essential to determine the extent of unroofing the aortic wall (14). In another study, Koshiyama et al. (15) reported an alternative technique, known as the Imai technique, which involves ASO without coronary relocation by creating an aortopulmonary fenestration. This technique is a preferred option for high-risk coronary anatomies such as intramural CA with distal stenosis and single-orifice coronary sinus, in addition, with superior long-term results compared to the double coronary button technique (15).

In our patient, the presence of significant risk factors such as CA pattern, juxta-commissural location of one ostium, and intramural course of the LAD/RCA coronary trunk and our inability to detect the presence of an intramural segment at the time of arterial switch led to coronary obstruction and myocardial ischemia postoperatively with severe ventricular dysfunction during the immediate post-operative period and required VA ECMO support. A multidisciplinary team involving cardiac surgery, cardiac imaging, and interventional and pediatric ICU allowed us, in spite of a complicated clinical course, to precisely identify the presence of CA obstruction and intervene successfully by unroofing the intramural portion of the CA and proceeding with extensive coronary arterioplasty. This approach successfully corrected the myocardial ischemia and lead to a positive outcome. With successful ASO, a long-term outcome among patients with intramural CA is favorable (14, 16).

We described a novel CA origin pattern in our index case. The presence of an unusual CA pattern, intramural course of CA, and CM can result in severe myocardial ischemia, increased morbidity, and mortality. A careful hemodynamic assessment and advanced cardiac imaging combined with a multidisciplinary approach are essential for a successful outcome including the need for assessment for unroofing the intramural portion of the CA.

## Data Availability Statement

The original contributions presented in the study are included in the article/supplementary material, further inquiries can be directed to the corresponding author/s.

## Ethics Statement

Ethical review and approval was not required for the study on human participants in accordance with the local legislation and institutional requirements. Written informed consent to participate in this study was provided by the participants' legal guardian/next of kin.

## Author Contributions

All authors listed have made a substantial, direct, and intellectual contribution to the work and approved it for publication.

## Conflict of Interest

The authors declare that the research was conducted in the absence of any commercial or financial relationships that could be construed as a potential conflict of interest.

## Publisher's Note

All claims expressed in this article are solely those of the authors and do not necessarily represent those of their affiliated organizations, or those of the publisher, the editors and the reviewers. Any product that may be evaluated in this article, or claim that may be made by its manufacturer, is not guaranteed or endorsed by the publisher.

## References

[1] Baraona F Valente AM Porayette P Pluchinotta FR Sanders SP. Coronary arteries in childhood heart disease: implications for management of young adults. J Clin Exp Cardiolog. (2012) S8:006. 10.4172/2155-9880.S8-006 24294539 PMC3842035

[2] Pasquali SK Hasselblad V Li JS Kong DF Sanders SP. Coronary artery pattern and outcome of arterial switch operation for transposition of the great arteries: a meta-analysis. Circulation. (2002) 106:2575–80. 10.1161/01.CIR.0000036745.19310.BB 12427654

[3] Metton O Calvaruso D Gaudin R Mussa S Raisky O Bonnet D. Intramural coronary arteries and outcome of neonatal arterial switch operation. Eur J Cardiothorac Surg. (2010) 37:1246–53. 10.1016/j.ejcts.2009.12.042 20153213

[4] Angelini P. Coronary artery anomalies: an entity in search of an identity. Circulation. (2007) 115:1296–305. 10.1161/CIRCULATIONAHA.106.618082 17353457

[5] Chiu IS Chu SH Wang JK Wu MH Chen MR Cheng CF. Evolution of coronary artery pattern according to short-axis aortopulmonary rotation: a new categorization for complete transposition of the great arteries. J Am Coll Cardiol. (1995) 26:250–8. 10.1016/0735-1097(95)00187-5 7797758

[6] Prêtre R Tamisier D Bonhoeffer P Mauriat P Pouard P Sidi D. Results of the arterial switch operation in neonates with transposed great arteries. Lancet. (2001) 357:1826–30. 10.1016/S0140-6736(00)04957-6 11410190

[7] Kim SJ Kim WH Lim C Oh SS Kim YM. Commissural malalignment of aortic-pulmonary sinus in complete transposition of great arteries. Ann Thorac Surg. (2003) 76:1906–10. 10.1016/S0003-4975(03)01068-3 14667609

[8] Al Nasef M Alghamdi MH Bello Valls ML Zahrani AM AlAkfash A Ardah HI. Commissural malalignment as a predictor of coronary artery abnormalities in patients with transposition of great arteries. J Congenit Heart Dis. (2020) 4:7. 10.1186/s40949-020-00039-7

[9] Villa AD Sammut E Nair A Rajani R Bonamini R Chiribiri A. Coronary artery anomalies overview: the normal and the abnormal. World J Radiol. (2016) 8:537–55. 10.4329/wjr.v8.i6.537 27358682 PMC4919754

[10] Topaz O DeMarchena EJ Perin E Sommer LS Mallon SM Chahine RA. Anomalous coronary arteries: angiographic findings in 80 patients. Int J Cardiol. (1992) 34:129–38. 10.1016/0167-5273(92)90148-V 1737663

[11] Bertail-Galoin C Leconte C Bakloul M Perouse-de-Montclos T Moulin-Zinsch A Martin-Bonnet C. Value of preoperative echocardiography for the diagnosis of coronary artery patterns in neonates with transposition of the great arteries. Arch Cardiovasc Dis. (2021) 114:115–21. 10.1016/j.acvd.2020.06.005 33069638

[12] Moll M Moll JA Moll JJ Łubisz M Michalak KW. Intramural coronary pattern in patients with transposition: incidence and impact on follow-up. Eur J Cardiothorac Surg. (2020) 58:145–52. 10.1093/ejcts/ezaa021 32057070

[13] Sun H Dun Y Yan J Yang K Hua Z Wang Q. Clinical outcome of patients with transposition of the great arteries and intramural coronary artery. Pediatr Cardiol. (2021) 42:417–24. 10.1007/s00246-020-02499-5 33591387

[14] Kim H Sung SC Kim SH Chang YH Ahn HY Lee HD. Arterial switch operation in patients with intramural coronary artery: early and mid-term results. Korean J Thorac Cardiovasc Surg. (2011) 44:115–22. 10.5090/kjtcs.2011.44.2.115 22263137 PMC3249286

[15] Koshiyama H Nagashima M Matsumura G Hiramatsu T Nakanishi T Yamazaki K. Arterial switch operation with and without coronary relocation for intramural coronary arteries. Ann Thorac Surg. (2016) 102:1353–9. 10.1016/j.athoracsur.2016.03.031 27209612

[16] Wong SH Finucane K Kerr AR O'Donnell C West T Gentles TL. Cardiac outcome up to 15 years after the arterial switch operation. Heart Lung Circ. (2008) 17:48–53. 10.1016/j.hlc.2007.06.523 17669687

